# Sequence analysis of *MYOC* and *CYP1B1* in a Chinese pedigree of juvenile glaucoma with goniodysgenesis

**Published:** 2009-08-07

**Authors:** Xiaoming Chen, Naihong Yan, Hongmin Yun, Jingjing Sun, Man Yu, Jiumo Zhou, Guiqun Cao, Hongbo Yin, Mao Li, Xuyang Liu

**Affiliations:** Ophthalmic Laboratories and Department of Ophthalmology, West China Hospital, Sichuan University, Chengdu, P. R. China

## Abstract

**Purpose:**

This study was designed to analyze two candidate genes, myocilin (*MYOC*) and cytochrome P450 1B1 (*CYP1B1*), in a Chinese pedigree of juvenile glaucoma with goniodysgenesis.

**Methods:**

In a three-generation family of juvenile glaucoma with goniodysgenesis (13 members), six of them were patients with glaucoma and the rest were asymptomatic. All members of the family underwent complete ophthalmologic examinations. Exons of *MYOC* and *CYP1B1* were amplified by polymerase chain reaction, sequenced, and compared with a reference database.

**Results:**

Elevated intraocular pressure (IOP) and visual function impairment was found in all patients, and goniodysgenesis was noticed in five of them (nine eyes) with relatively transparent corneas. One *MYOC* heterozygous mutation, c.1109 C>T (P370L), in exon 3 was identified in all six patients but not in the asymptomatic family members. Two *CYP1B1* single nucleotide polymorphisms (SNPs), g.3947 C>G (R48G) in exon 2 and 372−12 C>T in intron 1, were identified in all six patients and but not in the asymptomatic family members except the proband’s grandmother. Three SNPs were identified, 730 + 35 A>G in intron 2 of *MYOC* and g.8131 G>C (V432L) and g.8184 T>C (D449D) in exon 3 of *CYP1B1*.

**Conclusions:**

The presence of a P370L mutation of *MYOC* in all six glaucoma patients suggests a casual association between this mutation and juvenile glaucoma with goniodysgenesis. The possible role of SNPs of *CYP1B1* in the pathogenesis of the disease remains to be elucidated.

## Introduction

Unlike primary open-angle glaucoma (POAG) or juvenile open-angle glaucoma (JOAG), juvenile glaucoma can be associated with goniodysgenesis, a maldevelopment of the iridocorneal angle of the eye [1,2]. If intraocular pressure (IOP) becomes elevated after the age of three (late congenital glaucoma), the patients may not present symptoms such as buphthalmos, lacrimation, photophobia, and blepharospasm.

Genetic factors play a major role in the pathogenesis of glaucoma, but the molecular basis still remains unknown. To date, about 23 loci have been linked to different types of glaucoma, and four genes, myocilin (*MYOC*), optineurin (*OPTN*), cytochrome P450 1B1 (*CYP1B1*), and WD repeat domain 36 (*WDR36*), have been identified as glaucoma causing genes [3-6]. MYOC is a secreted glycoprotein normally expressed in several ocular and non-ocular tissues. To date, more than 70 mutations have been detected in *MYOC* in different population groups, contributing to approximately 3% of familial autosomal dominant adult-onset open-angle glaucoma and a greater proportion of JOAG. Most of the mutations were located in exon 3 where the olfactomedin-like domain is located [7]. Recently, mutations in *MYOC* have been linked to primary congenital glaucoma in India and China [8-10].

*CYP1B1* has been shown to cause primary congenital glaucoma (PCG), JOAG, and POAG [5,11-13]. It is thought that *CYP1B1* participates in the development of trabecular meshwork (TM) of the eye [12]. *CYP1B1* consists of three exons of which only exons 2 and 3 encode the protein. So far, certain sequence variances in *CYP1B1* such as g.3947 C>G (R48G) in exon 2 and 372−12 C>T in intron 1 have been identified as single nucleotide polymorphisms (SNPs) based on sequence analyses [14].

In this study, alterations in *MYOC* were analyzed, and a known mutation, which was segregated with the disorder within the family and appeared to be the disease-causing gene, was found. However, this still cannot explain the goniodysgenesis predominantly seen in patients of this family. We therefore investigated *CYP1B1*, a gene known to be related to the development of the iridocorneal angle [11]. Mutations in both *MYOC* and *CYP1B1* have been found in the juvenile or early-onset glaucoma pedigree. These mutations are different from what we first reported in the study of a juvenile glaucoma pedigree with goniodysgenesis, although these mutations were previously reported in POAG and PCG.

## Methods

### Family recruitment and clinical examination

A three-generation family with juvenile glaucoma with goniodysgenesis was recruited from the clinic of the Department of Ophthalmology at West China Hospital (Sichuan University, Chengdu, P. R. China). The study was approved by the medical ethics committee of the West China Hospital of Sichuan University. This study adhered to the tenets of the Declaration of Helsinki. All subjects were clinically evaluated by glaucoma specialists and diagnosed with juvenile glaucoma with goniodysgenesis.

**Figure 1:**
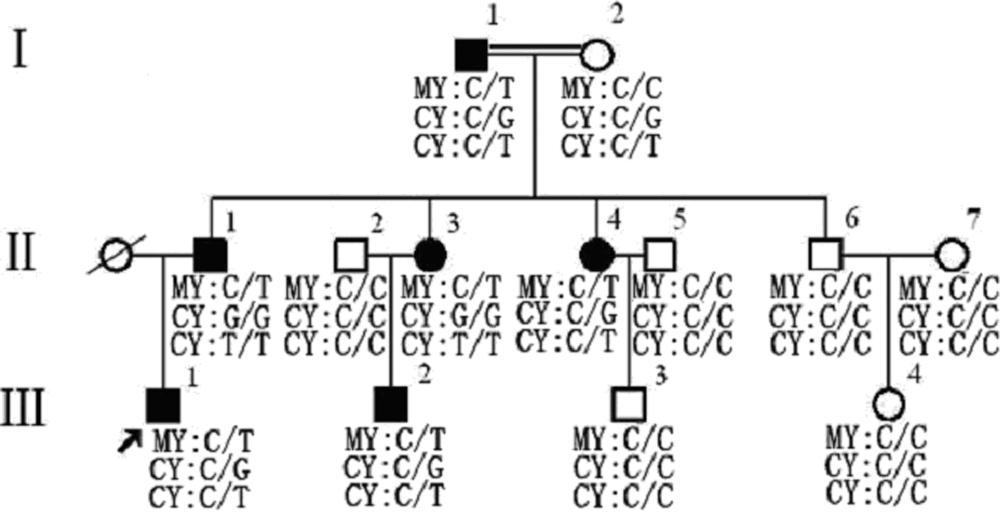
Pedigree of the Chinese glaucoma family. The variations of *MYOC* and *CYP1B1* genes were summarized in this figure of the Chinese family segregating juvenile glaucoma with goniodysgenesis. The *MYOC* heterozygous mutation, C>T (P370L) in exon 3 was identified in all six patients but not in asymptomatic family members. Two* CYP1B1* SNPs, C>G (R48G) in exon 2 and C>T in intron 1, were identified in all six patients and but not in asymptomatic family members except the proband’s paternal grandmother. Abbreviations: MY, *MYOC*; CY, *CYP1B1*. Arrow indicates the proband. (III:1).

Juvenile glaucoma with goniodysgenesis belongs to developmental glaucoma, which was defined as meeting all of the following criteria: goniodysgenesis with secondary causes (e.g., trauma, uveitis, or steroid-induced glaucoma) excluded; open anterior chamber angle; IOP greater than 21 mmHg in both eyes; and characteristic glaucomatous optic disc damage and/or visual field loss. The pedigree structure of the Chinese family with summaries of the variations in *MYOC* and *CYP1B1* is described in Figure 1.

### DNA extraction

Genomic DNA was extracted from 200 μl whole blood using a Qiamp Blood Kit (Qiagen, Hilden, Germany). All the procedures were performed according to the manufacturer’s protocol. DNA integrity was evaluated by 1% agarose gel electrophoresis.

### PCR amplification

Intronic primers flanking the exons (Table 1) were designed based on gene sequences of *MYOC* (GenBank AF001620) and *CYP1B1* (GenBank U56438) and synthesized by Invitrogen (Carlsbad, CA). DNA fragments were amplified by polymerase chain reaction (PCR) using a MyCycler thermocycler (Bio-Rad, Hercules, CA). The 30 μl PCR reaction mixture included 30 ng DNA, 1× PCR buffer, 2.5 mM MgCl_2_, 0.3 mM of each of dNTPs, 1.5 U Pfu DNA polymerase, and 1.0 μM each of the forward and reverse primers. All reagents used in this procedure were purchased from MBI (MBI Fermentas, Vilnius, Lithuania). The reactions were incubated in a 96 well plate at 95 °C for 2 min followed by 30 cycles at 94 °C for 10 s, 54 °C–56 °C for 30s, and 72 °C for 1–2 min and then a final extension at 72 °C for 5 min.

**Table 1:** Primers used in PCR for amplification of *MYOC* and *CYP1B1*.

**Exons**	**Primer sequence (forward/reverse)**	**Product size (bp)**
*MYOC* 1	5′-TCACCAAGCCTCTGCAATG-3′	654
	5′-TGAACTCAGAGTCCCCCCAC-3′	
*MYOC* 2	5′-GCACCTGCCACCACATCC-3′	657
	5′-ACACATCTCTCATCTTGCCC-3′	
*MYOC* 3	5′-GCTGTCACATCTACTGGCTC-3′	927
	5′-ATCTCCTTCTGCCATTGCCT-3′	
*CYP1B1* 2	5′-CATTTCTCCAGAGAGTCAGC-3′	1260
	5′-GCTTGCAAACTCAGCATATTC-3′	
*CYP1B1* 3	5′-ACCCAATGGAAAAGTCAGCC-3′	927
	5′-GCTTGCCTCTTGCTTCTTATT-3′	

The first column shows the sequenced exons of *MYOC* and *CYP1B1*; the second column shows the primer sequences, both forward and reverse ones, used to amplify the corresponding exons indicated in the first column; the third column shows the expected size of the PCR products.

### ﻿Sequencing and data analysis

PCR products were directly sequenced using an ABI 377XL automated DNA sequencer (Applied Biosystems, Foster City, CA). Sequence data were compared pair-wise with the published *MYOC* and *CYP1B1* sequences.

## Results

### The proband

**Figure 2:**
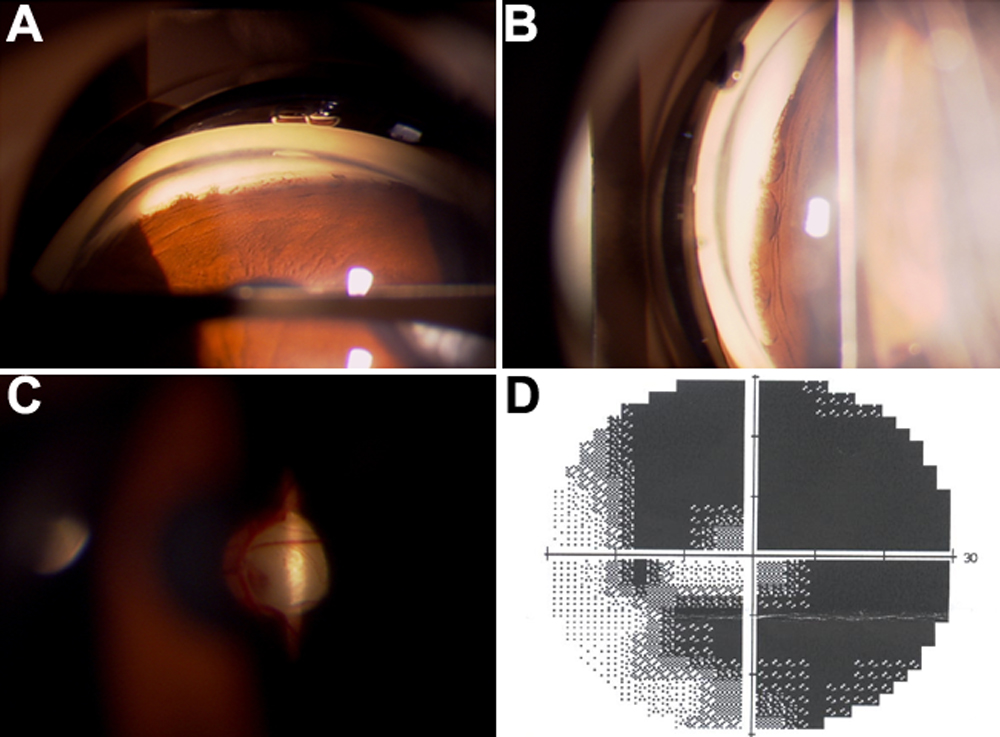
Clinical manifestations of the proband. **A** and **B** showed the maldevelopment of the anterior chamber angle. **C** and **D** showed glaucomatous optic disc atrophy and visual field defects, respectively.

The proband (III:1) was diagnosed with glaucoma (late stage, both eyes) at the age of 16 years. A cup-disc ratio of 0.90–1.0, elevated IOP (42 mmHg in the right eye and 34 mmHg in the left eye), and goniodysgenesis as described below were observed. No other ocular abnormalities or systemic disorders were found. Trabeculectomy was performed for both eyes. Three years after surgery, a cup-disc ratio of 0.95–1.0, open grade III (Shaffer) angles, and late-stage glaucomatous visual field loss were noticed in both eyes (Figure 2). His developmental angle anomaly was characterized by the absence of the angle recess in some area and the presence of iris tissue inserted either flat or concave shaped directly into the trabecular meshwork all over the iridocorneal angle.

### Other affected family members

The IOP of the affected members including the proband in this family varied between 25 mmHg and 55 mmHg, depending on different treatment situations (no treatment, topical antiglaucoma medications, and/or surgeries) for each individual. The proband’s father (II:1) was diagnosed with glaucoma at 24 years of age. Six years after the diagnosis, he became blind in both eyes. The father’s two younger sisters (II:3 and II:4) were affected, but his younger brother (II:6) was normal. The proband’s mother did not have any ocular disorders, and she did not recall any glaucoma cases among her lineage or relatives. The proband’s paternal grandfather (I:1) and grandmother (I:2) had a consanguineous marriage. The grandfather (I:1) has had glaucoma since he was young, and the grandmother (I:2) never had glaucoma or other noticeable ocular disorders.

Goniodysgenesis as described in the proband was noticed in four of his relatives with relatively transparent corneas. None of the affected individuals showed an enlarged cornea, excessive tearing, or photophobia. Table 2 is a summary of the major clinical findings and the variations in *MYOC* and *CYP1B1* of the affected members in this family.

**Table 2:** Clinical findings in glaucoma patients in this the family.

**Subject**	**Sex/Age (years)**	**Highest IOP (mmHg) Observed (OD/OS)**	**Cup-disc ratio (OD/OS)**	**Visual acuity (OD/OS)**	**Trabeculodysgenesis**	**Mutation**	**SNPs**	
						***MYOC* P370L**	***CYP1B1* R48G**	***CYP1B1* C>T**
III:1	M/26	28/23	1.0/0.95	0.1/0.5	yes/yes	het	het	het
III:2	M/22	25/44	1.0/1.0	NLP/NLP	yes/yes	het	het	het
II:1	M/41	52/NA	1.0/inv	NLP/NLP	yes/inv	het	hom	hom
II:3	F/44	NA/50	inv/inv	NLP/NLP	inv/inv	het	hom	hom
II:4	F/39	53/57	inv/inv	NLP/NLP	yes/yes	het	het	het
I:1	M/71	34/30	1.0/1.0	NLP/ FC	yes/yes	het	het	het

The clinical information of the glaucoma patients in this pedigree were shown in this table, including gender, age, highest IOP, cup-disc ratio, visual acuity, trabeculodisgenesis and sequence variations found in *MYOC* and *CYP1B1*. In the table, abbreviations are: M, male; F, female; OD, right eye; OS, left eye; IOP, intraocular pressure; NLP, no light perception; FC, Figure counting; inv, invisible due to cornea opacification and/or cataract; het, heterozygous; hom, homozygous.

### Asymptomatic family members

The proband’s paternal grandmother (I:2) was 65 years old, and her visual acuity was 20/25 with corrections. The visual acuities of the proband’s paternal uncle (II:6) who was the only unaffected person in the second generation and of the other two unaffected individuals (III:3 and III:4) were 20/20. The IOP, anterior chamber angle, and the cup-disc ratio of all asymptomatic members were in normal ranges.

### Sequencing results

The mutations that cosegregated with the disorder within the family are summarized in Figure 1 and Table 2.

#### MYOC

**Figure 3:**
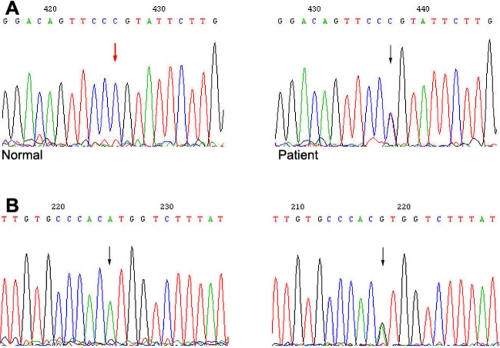
*MYOC* mutation and variation in the glaucoma family. **A**: Heterozygous C>T **(**P370L) mutation of *MYOC* is shown. **B**: A>G variation of *MYOC* is shown. Arrows indicate the sequence difference, and the one in red indicates the wild type sequence.

Sequence analysis of *MYOC* revealed a heterozygous mutation, C>T **(**P370L), in exon 3 in all patients but not in any of the asymptomatic members of the family. The P370L *MYOC* mutation cosegregated with the disorder within the family. And one SNP of *MYOC* was identified in intron 2 (A>G; Figure 3).

#### CYP1B1 

**Figure 4:**
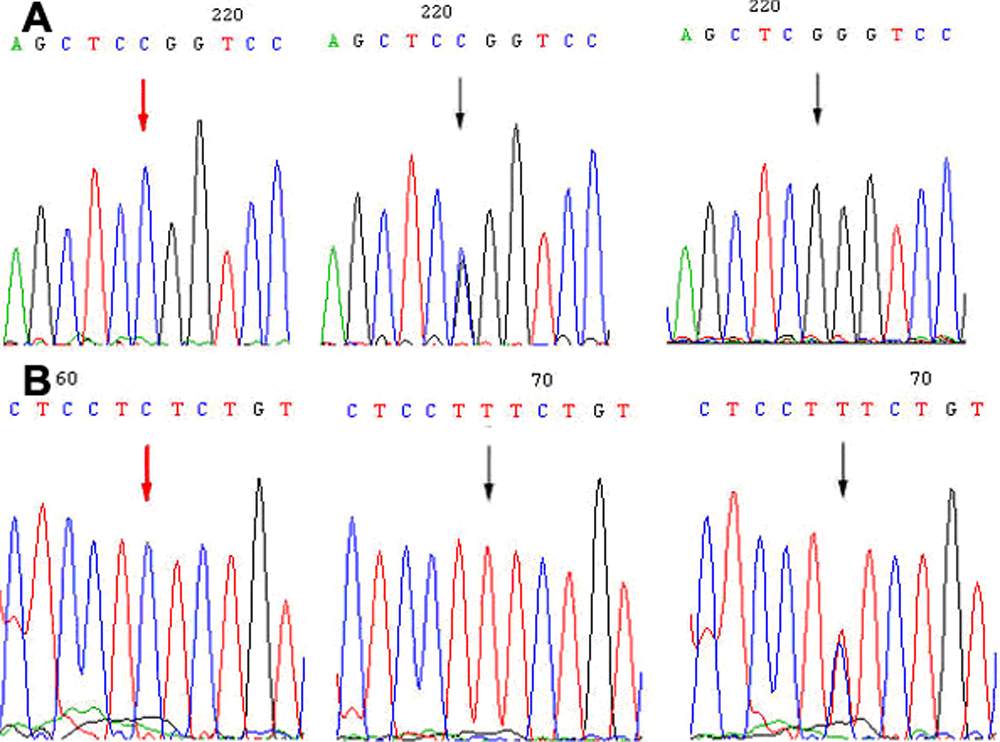
Two *CYP1B1* SNPs in the glaucoma family. **A**: C>G variation in exon 2 of *CYP1B1*. The codon CGG in exon 2 was replaced by GGG, resulting in a change of amino acid from arginine to glycine (R48G). **B**: C>T variation in intron 1 of *CYP1B1*. These two SNPs occurred in all six patients afflicted with glaucoma, and were not found in asymptomatic family members except the proband’s paternal grandmother. Arrows indicate the sequence difference, and the one in red indicates the wild type sequence.

**Figure 5:**
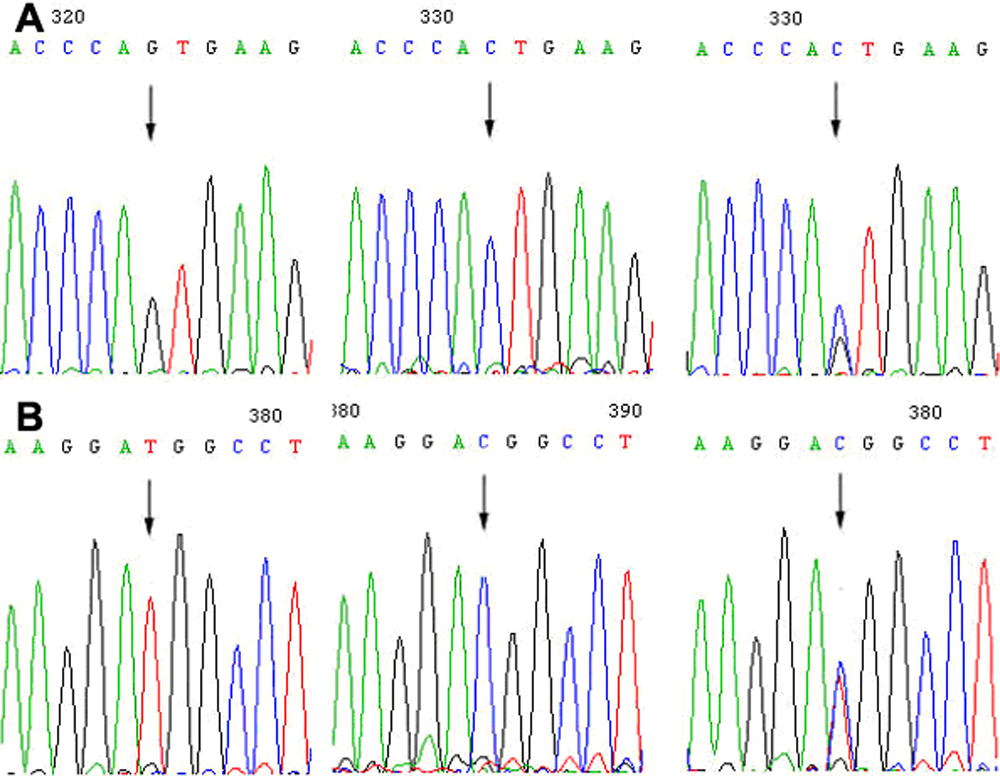
Two *CYP1B1* variations in the glaucoma family. **A**: G>C (V432L) variation in exon 3 of *CYP1B1*. **B**: T>C (D449D) variation in exon 3 of *CYP1B1*. Arrows indicate the sequence difference.

Two *CYP1B1* SNPs, C>G (R48G) in exon 2 and C>T in intron 1, were identified. These two SNPs occurred in all six patients afflicted with glaucoma (four were heterozygous and two were homozygous) and were not found in any of the asymptomatic family members except the proband’s grandmother (Figure 4). Two SNPs were identified, G>C (V432L) and T>C (D449D) both in exon 3 of *CYP1B1* (Figure 5).

## Discussion

The terms congenital, developmental, infantile, and juvenile glaucoma appear to be overlapping and confusing. Because of a variation in the severity of angle abnormalities, glaucoma may appear early or late in life. Therefore, it may not be appropriate to decide the classification or the mode of inheritance of the glaucoma based on the patient's age at the onset of the symptoms when angle abnormality is the common etiologic factor [1]. A child with primary glaucoma but without angle abnormalities should be diagnosed as JOAG. Any childhood glaucoma caused by maldevelopment of the iridocorneal angle is considered primary congenital glaucoma, which is usually characterized by buphthalmos, lacrimation, photophobia, and blepharospasm. For the affected patients in this pedigree, these symptoms were not noticed by either themselves or by ophthalmologists at the first visit. No evidence is available for the exact ages when they had glaucoma, but the onset of glaucoma is likely much earlier than the time they noticed having eye problems. For example, one of the patients (III:2) mentioned that he had “high myopia that got worse very fast” when he was in primary school. Cornea damage occurred in some of the patients probably due to long-term uncontrolled high IOP. A gonioscopic examination was performed for those eyes with relatively transparent cornea, and all of them showed developmental iridocorneal angle anomaly.

More than 70 mutations have been identified in *MYOC* of POAG patients from India, England, France, North America, Japan, and Germany, indicating that different *MYOC* mutations may contribute to the pathogenesis of POAG in populations of different ethnicity [15-22]. *MYOC* mutations were also found in a few cases of sporadic PCG [8,23]. Zhou et al. [24] reported the homozygous mutation in intron 2 of *MYOC* in one family with both PCG and POAG in China. In that pedigree, one was diagnosed as a PCG patient who developed symptoms of epiphora, photophobia, edematous, enlarged cornea, and increased ocular axial length while the anterior chamber angle was found open and “normal”, and no maldevelopment of the iridocorneal angle was noticed [24]. In our study, *MYOC* was screened for mutations in the pedigree with juvenile glaucoma with goniodysgenesis. P370L as a heterozygous mutation was identified in *MYOC* in the affected members but not in the normal individuals, and the mutation cosegregated with the disorder within the pedigree, suggesting that *MYOC* may be the glaucoma-causing gene in this family.

However, the *MYOC* mutation does not support a role of this gene in the maldevelopment of the iridocorneal angle. Therefore, another candidate gene, *CYP1B1*, was screened. Previous studies have identified *CYP1B1* as a causative gene in PCG and occasionally as a causative gene in POAG as well as several anterior segment dysgenesis disorders [25]. Furthermore, *CYP1B1*-deficient mice exhibit abnormalities in their ocular drainage structure and trabecular meshwork (TM), which are similar to those reported in human PCG patients [26]. It was speculated that the CYP1B1 protein might be involved in the generation of a regulatory molecule that controls the expression of genes involved in the development of the anterior chamber angle [25]. In this pedigree, the R48G variation in exon 2 and C>T variation in intron 1 of *CYP1B1* were found in all six patients and not in any of the asymptomatic family members except the proband’s grandmother. We tried to compare the phenotypes between the two patients homozygous for the *CYP1B1* variation and the others heterozygous for these variations. The former did show a more severe and complicated phenotype such as cataract. However, both of them received less antiglaucoma treatments, which may explain the severity of the disorder.

Studies by Hollander et al. [27] have indicated that multiple *CYP1B1* variances in which the R48G was not included may be associated with severe or moderate goniodysgenesis in congenital glaucoma. In our pedigree study, individuals carrying both the *MYOC* mutation and *CYP1B1* variation were affected with juvenile glaucoma with goniodysgenesis. However, it is still unlikely that the combination of *MYOC* and *CYP1B1* variations correlate with an earlier manifestation of the disease. The *CYP1B1* variation described in this study, C>G (R48G) in exon 2, has not been reported to be associated with goniodysgenesis, and it was identified as an SNP previously. Stoilov IR et al. [14] have reported that the R48G variation frequency was 0.29 in normal populations in the UK and Turkey. It was also reported that R48G variation frequency was 0.295 and 0.205 in patients with primary congenital glaucoma and normal populations, respectively, in Brazil, and the frequency variation was 0.435 and 0.39 in patients of JOAG and normal populations, respectively, in India [28,29]. Therefore, this variance identified in this pedigree may just have coincidentally occurred in six patients. This is supported by the fact that this variance was also found in the proband’s grandmother who was asymptomatic.

In conclusion, the P370L mutation of *MYOC* in this pedigree appears to be the cause of the disease. However, it remains unclear the gene defect underlying goniodysgenesis of the six patients with both a *MYOC* mutation and *CYP1B1* variance. Previous studies indicated that *CYP1B1* may act as a modifier of *MYOC* expression and that the two genes may interact via a common pathway [11,30]. Obviously, further studies are needed to elucidate the possible role of *CYP1B1* variations such as R48G in juvenile glaucoma with goniodysgenesis.
